# Heterogeneity of primary outcome measures used in clinical trials of treatments for intermediate, posterior, and panuveitis

**DOI:** 10.1186/s13023-015-0318-6

**Published:** 2015-08-19

**Authors:** Alastair K. Denniston, Gary N. Holland, Andrej Kidess, Robert B. Nussenblatt, Annabelle A. Okada, James T. Rosenbaum, Andrew D. Dick

**Affiliations:** Institute of Translational Medicine, Birmingham Health Partners, University of Birmingham, Birmingham, UK; Department of Ophthalmology, Queen Elizabeth Hospital Birmingham, University Hospitals Birmingham NHS Foundation Trust, Birmingham, UK; UCLA Stein Eye Institute and the Department of Ophthalmology, Ocular Inflammatory Disease Center, David Geffen School of Medicine at UCLA, 100 Stein Plaza, UCLA, Los Angeles, CA 90095-7000 USA; National Eye Institute, National Institutes of Health, Bethesda, Maryland USA; Department of Ophthalmology, Kyorin University School of Medicine, Tokyo, Japan; Department of Ophthalmology, Casey Eye Institute, Oregon Health & Science University, USA Legacy Devers Eye Institute, Portland, Oregon USA; Bristol Eye Hospital, University Hospitals Bristol NHS Foundation Trust, Bristol, UK; Academic Unit of Ophthalmology, University of Bristol, Bristol, UK; NIHR Biomedical Research Centre for Ophthalmology, Moorfields Eye Hospital NHS Foundation Trust and UCL Institute of Ophthalmology, London, UK

**Keywords:** Uveitis, Clinical trials, Outcome measures, Endpoints, Composite endpoints

## Abstract

**Background:**

Uveitis describes a heterogeneous group of conditions characterized by intraocular inflammation. Since most of the sight-threatening forms of uveitis are individually rare, there has been an increasing tendency for clinical trials to group distinct uveitis syndromes together despite clear variations in phenotype which may reflect real aetiological and pathogenetic differences. Furthermore this grouping of distinct syndromes, and the range of manifestations within each uveitis syndrome, leads to a wide range of possible outcome measures. In this study we wished to review the degree of consensus or otherwise in the choice of primary outcome measures for registered clinical trials related to uveitis.

**Methods:**

Systematic review of data provided in clinical trial registries describing clinical trials dealing with medical treatment of intermediate, posterior, or panuveitis through 01 October 2013. We reviewed 15 on-line clinical trial registries approved by the International Committee of Medical Journal Editors. We identified all that met the following inclusion criteria: prospective, interventional design; target populations with intermediate, posterior or panuveitis; and one or more pre-specified outcome measures that were related to uveitis. Primary outcome measures were classified in terms of type (efficacy or safety or both; single, composite, or multiple); dimension (disease activity, disease damage, measured or patient-reported visual function); and domain (the specific study variable being measured).

**Results:**

Of 195 registered uveitis studies, we identified 104 clinical trials that met inclusion criteria. There were 14 different domains used as primary outcome measures. Among clinical trials that utilized primary outcome measures of treatment efficacy (*n* = 94), 70 (74 %) used a measure of disease activity (vitreous haze in 40/70 [57 %]; macular oedema in 19/70 [27 %]) and 49 (70 %) used a measure of visual function (visual acuity in all cases). Multiple primary outcome measures were used in 23 (22 %) of 104 clinical trials. With regard to quality, in 12 (12 %) of 104 clinical trials, outcome measures were poorly defined. No clinical trial utilized a patient-reported study variable as primary outcome measure.

**Conclusions:**

This systematic review highlights the heterogeneity of outcome measures used in recent clinical trials for intermediate, posterior, and panuveitis. Current designs prioritize clinician-observed measures of disease activity and measurement of visual function as outcome measures. This apparent lack of consensus regarding outcome measures for the study of uveitis is a concern, as it prevents comparison of studies and meta-analyses, and weakens the evidence available to stake-holders, from patients to clinicians to regulators, regarding the efficacy and value of a given treatment.

## Introduction

Uveitis describes a heterogeneous group of conditions characterized by intraocular inflammation. Most uveitis syndromes are individually rare, but for taxonomic and clinical convenience are commonly grouped according within an anatomical classification as being anterior, intermediate, posterior, or pan-uveitis [1–7]. The most sight-threatening forms are those that affect the more posterior structures of the eye – intermediate, posterior and pan-uveitis These three anatomical categories of uveitis often share the need for similar therapeutic strategies (usually systemic drug treatment) and are commonly grouped together in clinical trials, despite the wide range of systemic disease associations and clinical syndromes they represent. Intermediate, posterior and panuveitis each have an estimated prevalence of around 5-10/100 000 in Europe and 13-25/100 000 in the USA [1–6]. Evidently the individual syndromes are much rarer with over 30 definable uveitis syndromes, many of which may be classed as ‘very rare’ with a prevalence of less than or equal to 1 per 100 000 [3–6]. Examples include Sympathetic Ophthalmia, Birdshot Chorioretinopathy, Acute Posterior Multifocal Placoid Pigment Epitheliopathy and Serpiginous Choroidopathy. It should be noted that although individual uveitis syndromes are rare, they are collectively an important cause of vision loss, believed to account for 15 % of total blindness in the western world [1–3].

Research into the treatment of uveitis faces a number of challenges, with few randomized controlled clinical trials (RCTs) undertaken, and even fewer that demonstrate treatment benefit [8]. There are wide-ranging practices amongst specialists in their approaches to the treatment of uveitis, with most specialists citing a lack of evidence to support treatment decisions [9]. The advent of new intravitreal therapies is providing even greater choice and uncertainty for patients and clinicians [10–12].

We have argued that a fundamental obstacle to successful clinical trials dealing with uveitis is the lack of high-quality outcome measures [8]. Currently, vitreous haze score, as defined by Nussenblatt and associates [13], is a disease activity surrogate endpoint that is accepted by the United States Food and Drug Administration (FDA) for clinical trials that it reviews. This score utilizes a subjective six-point (0, 0.5, 1, 2, 3, or 4+) ordinal scale of the cloudiness of the vitreous humor. It has the advantages of being non-invasive and widely available, but it has significant inter-observer variability [14, 15]. Consequently a two-step change has been required to be considered significant [14], which is challenging, as most uveitis falls within the lower grades (2+ or less). Success in these clinical trials requires a combination of a near-perfect drug (including large effect; effect in almost all recipients, despite subject heterogeneity; and an acceptable side-effect profile) and a near-perfect study (successful recruitment; minimal drop-out; and minimal errors or missing data) [8].

Furthermore, a lack of consensus over which outcome measure(s) to use, and how to measure them, results in disparity of study design which limits evidence synthesis and prevents the pooling of study data for meta-analysis. An ability to compare new results to other studies is often a key requirement of regulatory authorities and health funders when evaluating and licensing novel therapeutics, and in this regard the evidence-base for uveitis consistently falls short. But issues around consistency of outcomes and their reporting are not confined to uveitis; indeed, there is growing recognition of the cost of ‘research waste,’ in which issues, such as non-reporting or selective reporting of data, inappropriate end-point selection, and inadequate trial design, among other factors, all contribute to a scenario in which ‘billions of dollars in investment are wasted’ [16].

In light of these problems, we have investigated the spectrum of outcome measures used in uveitis clinical trials, particularly focusing on those trials dealing with intermediate, posterior, and panuveitis. This systematic review surveys all such therapeutic clinical trials registered in databases approved by the International Committee of Medical Journal Editors (ICMJE) through 01 October 2013. We present the primary outcome measures identified in all these studies, noting the relative use of single, composite, and multiple outcomes, and the heterogeneity of outcome selection. In addition, we assess these data in terms of their potential impact on the clinical trials environment within the subspecialty of uveitis. We believe that the challenges of outcome selection in uveitis trials may be relevant to other sectors of the rare disease community faced with designing clinical trials for patients with syndromes that are individually rare and which exhibit a wide range of clinically-relevant manifestations.

## Methods

### Identification of clinical trial registries

All clinical trial databases that were registered with, and approved by, the ICMJE as of 01 October 2013 were identified [http://www.icmje.org/recommendations/browse/publishing-and-editorial-issues/clinical-trial-registration.html ] Included were those listed on the World Health Organization (WHO) International Clinical Trials Registry Platform (ICTRP) [http://www.who.int/ictrp/network/primary/en/] and the United States National Institutes of Health (NIH) “clinicaltrials.gov” website, [https://clinicaltrials.gov ] which is a data provider to the WHO ICTRP. The ICMJE states that it endorses those registries that meet the following requirements: accessible to the public at no charge; open to all prospective registrants; managed by a not-for-profit organization; have a mechanism to ensure the validity of the registration data; are electronically searchable; and include a minimum 20-item trial registration dataset at the time of registration and before enrollment of the first participant (available at www.who.int/ictrp/network/trds/en/index.html ). In its September 2004 editorial, the ICMJE announced that it would not consider a trial for publication unless the clinical trial had been included in an approved registry. ICMJE requirements stipulating trial registration as a requisite for publication were announced in 2004 and implemented through 2005, but due to retrospective registration these registries also included a number of clinical trials extending back as far as 2001.

### Selection of clinical trials for review

**Fig. 1:**
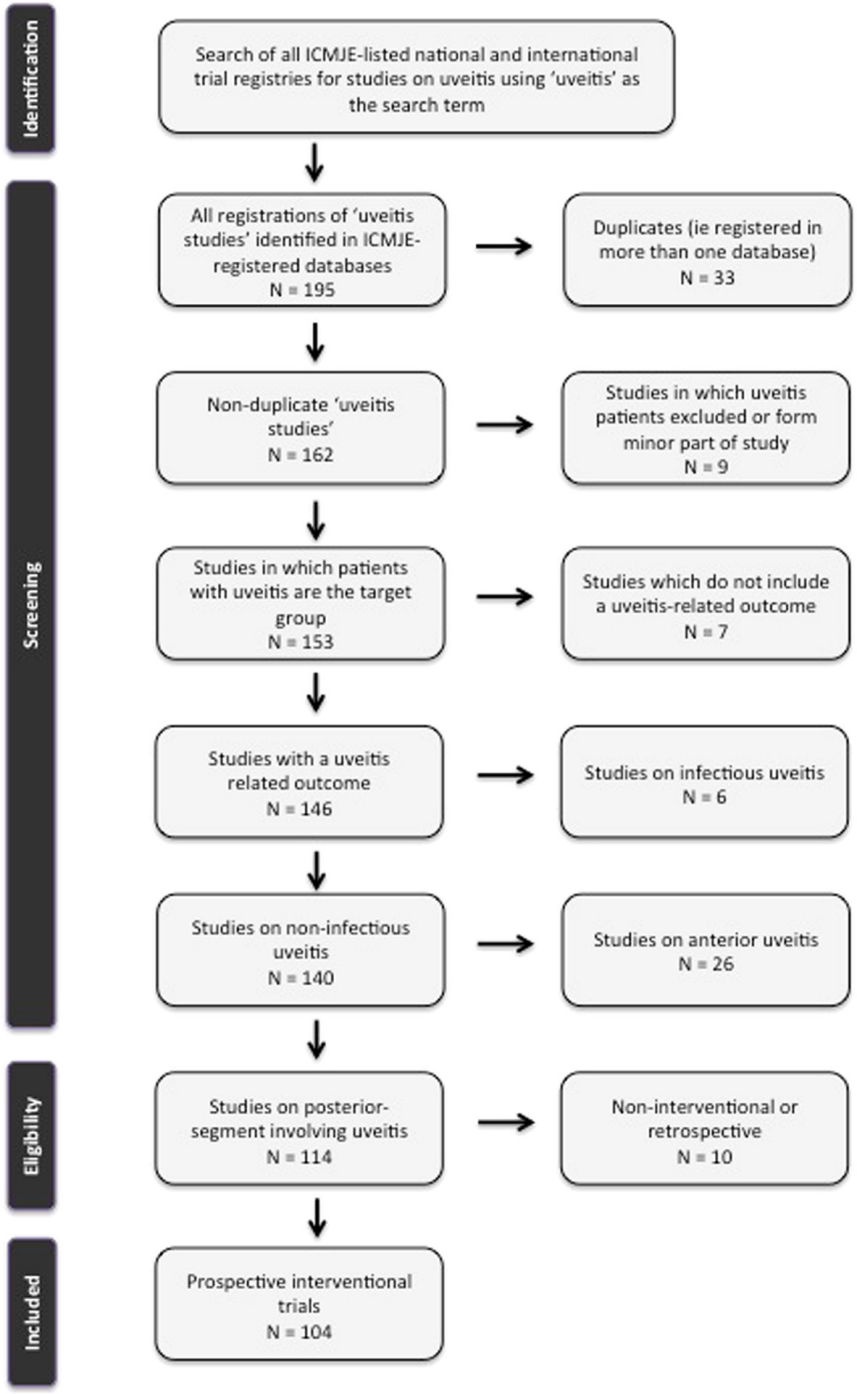
Flow chart depicting the process of identification, screening, and inclusion of uveitis clinical trials for this systematic review

We searched all identified registries for clinical trials related to uveitis, using the term “uveitis” within each registry’s electronic search capability, as a “free-text” search, a “key-word” search, or both, depending on the available options within the design of the registry. All registries were included regardless of language. Screening of identified clinical trials were then undertaken to remove the following types of studies, as shown in Fig. 1: duplicates; studies in which uveitis patients were not the target population; studies in which the primary outcome measure was not related to uveitis; studies of infectious uveitis; studies of anterior uveitis; and studies that were non-interventional or retrospective in design.

### Analysis of prospective interventional clinical trials

Studies that met inclusion criteria were analyzed according to their primary outcome measure. Outcome measures were transcribed verbatim from their register entry, then classified according to type, dimension, and domain. The type of outcome measure was described as being efficacy only, safety only, or both; and as being a single measure, a composite measure, or one of multiple, separate measures used to determine clinical trial results. A composite outcome measure was defined as one comprised of multiple study variables, any one of which could signal achievement of the outcome. For example, Suhler and associates described a composite outcome measure labeled “clinical success” comprised of the following study variables: visual acuity; control of intraocular inflammation; ability to taper concomitant medication therapy; and improvement in inflammatory signs on fluorescein angiography, ocular coherence tomography, or both. Clinical success was achieved when there was improvement in at least one study variable and worsening of none [17]. In contrast, “multiple outcomes” described those clinical trials in which more than one separate study variable was listed as a primary outcome measure, with apparent equal importance of each. and with no pre-specification of their contribution to a single composite score.

Dimension referred to whether the outcome measure described (1) disease activity (e.g. presence or absence of intraocular inflammatory signs and level of severity); (2) tissue damage attributable to the disease or other complications (e.g. cataract); (3) tested visual function (e.g. visual acuity; or (4) patient-reported visual function (e.g. NEI-VFQ-25 questionnaire). Domain described the specific study variable that was being measured (e.g. anterior chamber cell level or vitreous haze score for disease activity).

The quality of each outcome measure was assessed on the basis of definitions for each measure provided with registration information. Specifically, we sought descriptions about how study variables were to be categorized or quantified. Outcome measures were deemed to be poor quality, if, in the opinion of the reviewer, it would not be possible to reproduce results independently on the basis of the definitions provided.

## Results

### Identification of registries containing clinical trials relevant to uveitis

At the time of database search (01 October 2013), 15 registries were identified that were endorsed by the ICMJE. They included 11 national registries and four international/multinational registries; the latter being the International Standard Randomized Controlled Trial Number Registry (ISRCTN), the European Union Clinical Trials Register (EUCTR), the Pan African Clinical Trial Registry (PACTR), and ClinicalTrials.gov. Of the 11 national registries, 10 are “stand-alone’ sites, whereas the Japan Primary Registries Network represents a single portal to access three separate registries: the University Hospital Medical Information Network (UHMIN); the Japan Pharmaceutical Information Network (JAPIC); and the Japan Medical Association Center for Clinical Trials (JMACCT). Search of these databases identified one or more clinical trials containing reference to “uveitis” in 10 of 15 registries (eight of 11 national registries and three of four international registries, Table 1).

**Table 1:** Distribution of Trial Registrations. These figures include studies registered across multiple registries

Registry	Study Prefix	Total
Australian New Zealand Clinical Trial Registry	ACTRN	3
Brazilian Clinical Trials Registry	ReBec	0
Chinese Clinical Trial Register	ChiCTR	5
Clinical Trials Registry - India	CTRI	8
Cuban Public Registry of Clinical Trials	RPCEC	1
European Union Clinical Trials Register	EudraCT	43
German Clinical Trials Register	DRKS	0
International Standard Randomised Controlled Trial Number Register	ISRCTN	9
Iranian Registry of Clinical Trials	IRCT	4
Japan Primary Registries Network comprising:		
University Hospital Medical Information Network	UMIN	5
Japan Pharmaceutical Information Center	JAPIC	1
Japan Medical Association Center for Clinical Trials	JMACCT	0
Korea National Institute of Health Clinical Research Information Service	CRIS	0
Netherlands National Trial Register	NTR	4
Pan African Clinical Trial Registry	PACTR	0
Sri Lanka Clinical Trials Registry	SLCTR	0
USA National Institute of Health Clinicaltrials.gov	NCT	112
Total registrations		195

### Identification of interventional trials related to uveitis

Screening of the ICMJE-approved registries identified 195 clinical trials related to uveitis. After removal of duplicates, studies in which uveitis patients were not the target population, studies in which the primary outcome measure was not related to uveitis, studies of infectious uveitis, and studies of anterior uveitis, 113 studies were identified that were potentially relevant to this systematic review. In assessing eligibility by study design, nine studies were found to be non-interventional or retrospective, leaving 104 clinical trials for inclusion. Of these 104 clinical trials, 101 (97 %) involved a pharmacological agent, two (2 %) involved a surgical intervention, and one (1 %) involved a cell-based therapy.

### The Use of single, composite, and multiple primary efficacy outcome measures in reviewed clinical trials

All 104 included clinical trials had pre-identified primary outcome measures, which is a prerequisite for inclusion on ICMJE-approved registries. Primary outcome measures involved efficacy in 91 (88 %) clinical trials; safety in 10 (10 %) clinical trials; and both efficacy and safety in three (3 %) clinical trials. For those 94 clinical trials in which primary outcome measures involved efficacy, 36 (38 %) utilized a single efficacy variable (e.g. a two-step change in the NEI vitreous haze score); 35 (37 %) utilized a composite outcome (e.g. a score based on visual acuity, control of inflammation, tapering of medication therapy, and reduction of cystoid macular oedema [14]); and 23 (24 %) utilized multiple separate outcome measures, either all related to efficacy (20 clinical trials) or a mixture of efficacy and safety variables (3 clinical trials, Table 2).

**Table 2:** Types of Primary Outcome selected for use in Registered Trials for Posterior Segment Involving Uveitis (PSIU)

Type of Primary Outcomes	Design	Number (n/104)	Percentage (%)
Efficacy Outcome(s) Alone	Single efficacy outcome	36	35
	Composite outcomes	35	34
	Multiple efficacy outcomes	20	19
	All	91	88
Safety Outcome Alone	All	10	10
Mixed efficacy and Safety Outcomes	Composite efficacy outcome with safety outcome	0	0
	Multiple efficacy outcome with safety outcome	3	3
	All	3	3

Of the 94 clinical trials in which primary outcome measures involved efficacy, 75 included a broad range of disorders (intermediate, posterior, and panuveitis), whereas 19 were narrower in scope (18 involved only a single disorder; one included two disorders). Of the 35 clinical trials that used a composite outcome measure, 28 included multiple categories of uveitis, whereas 7 were disorder-specific (5 involving Behcet disease; one involving intermediate uveitis; and one involving multifocal choroiditis with panuveitis syndrome).

### Analysis of Pre-identified primary efficacy outcome measures by dimension and domain

The 94 clinical trials that included one or more efficacy outcome measures as the pre-specified primary outcome measure were analysed further in terms of dimension and domain. Overall 70 (74 %) of 94 clinical trials included one or more measures of disease activity as a primary outcome measure; 49 (52 %) included one or more measures of visual function (e.g. visual acuity) as a primary outcome measure; and 4 (4 %) included one or more measures of tissue damage or other disease complications as a primary outcome measure. No studies included a measure of patient reported visual function as a primary outcome measure. This prioritization of disease activity, followed by visual function performance, and then disease damage that we observed was not affected by whether the registration pre-specified a primary outcome measure involving a single efficacy variable; a composite outcome measure; or multiple outcome measures (Tables 3, 4 and 5).

**Table 3:** Primary Outcomes classified by Dimension and Domain: Single Efficacy Outcome Dataset

Dimension	Domain	Number (n/36)	Percentage (%)
Activity	All	18	50
	Vitreous haze	8	22
	Macular Oedema	8	22
	Treatment requirement	2	6
Damage/Other complications of disease	All	4	11
	Hypotony	2	6
	Elevated IOP	1	3
	Choroidal neovascular membrane	1	3
Visual function (performance)	All	14	39
	Visual acuity	14	39
Visual function (patient reported)	All	0	0
Unspecified		0	0

**Table 4:** Primary Outcomes classified by Dimension and Domain: Composite Efficacy Outcome Dataset

Dimension	Domain	Number (n/35)	Percentage (%)
Activity	All	32	91
	AC cells	21	60
	Vitreous haze	30	86
	Vitreous cells	2	6
	Snowballs	1	3
	Macular Oedema	4	11
	Chorioretinal inflammatory lesions	5	14
	Retinovascular inflammation	7	20
	Treatment requirement	10	29
Damage/Other complications of disease	All	0	0
Visual function (performance)	All	22	63
	Visual acuity	22	63
Visual function (patient reported)	All	0	0
Unspecified		3	9

**Table 5:** Primary Outcomes classified by Dimension and Domain: Multiple Efficacy Outcome Dataset

Dimension	Domain	Number (n/23)	Percentage (%)
Activity	All	20	87
	AC cells	4	17
	AC flare	2	9
	Vitreous haze	2	9
	Vitreous cells	1	4
	Snowballs	1	4
	Macular Oedema	7	30
	Chorioretinal inflammatory lesions	2	9
	Retinovascular inflammation	3	13
	Treatment requirement	4	17
	Unspecified ‘activity’	9	39
Damage/Other complications of disease	All	4	17
	Elevated IOP	3	13
	Cells on IOL	1	4
	Posterior capsular opacification	1	4
Visual function (performance)	All	13	57
	Visual acuity	13	57
Visual function (patient reported)	All	0	0
Unspecified		0	0

**Fig. 2:**
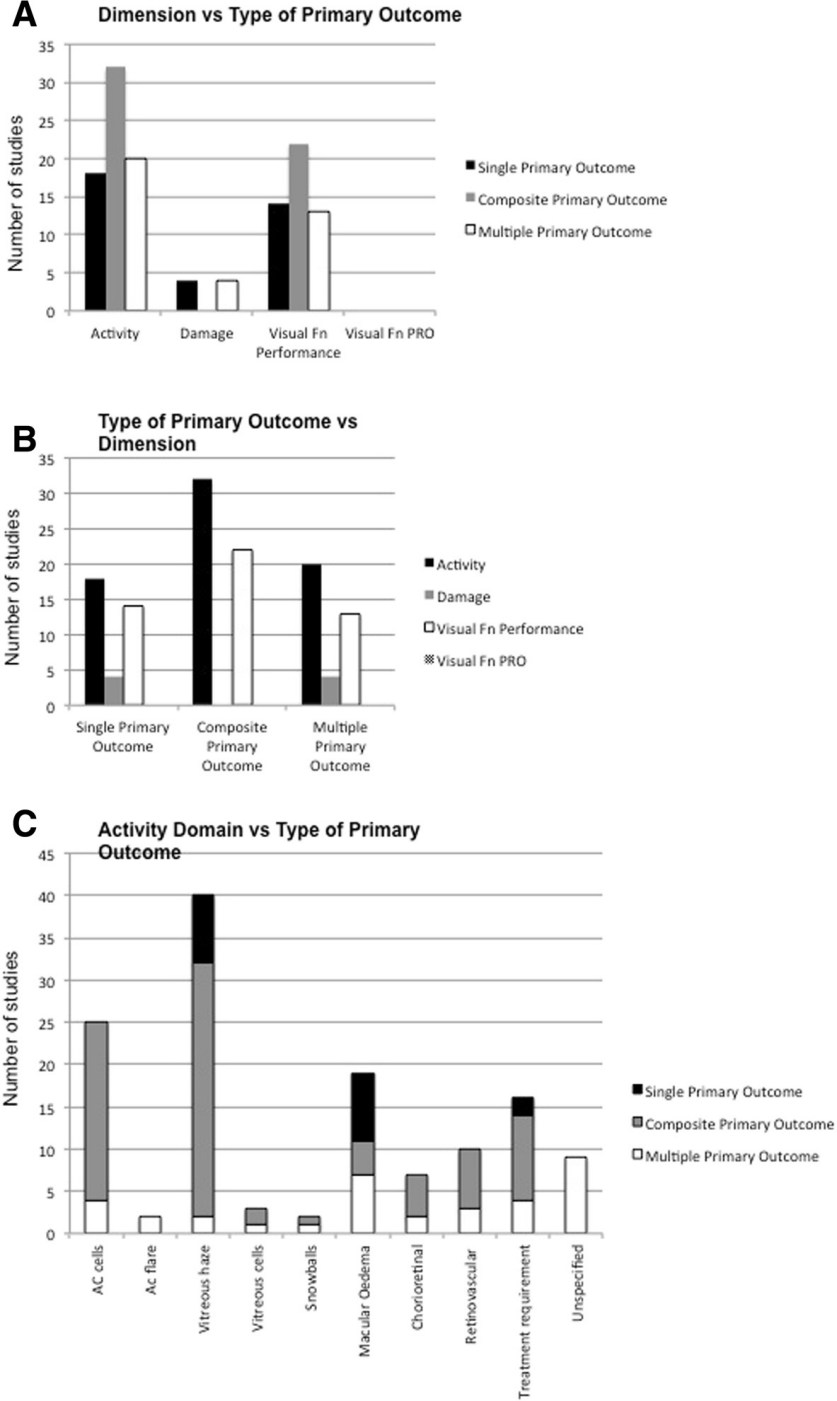
Column graphs depicting domains and selected dimensions used in pre-specified primary outcome measures of efficacy in uveitis clinical trials. **a, b** Dimensions according to whether primary outcome measure was single, multiple, or composite. **c** Activity domains, according to whether primary outcome measure was single, multiple, or composite

Domain selection varied according to whether the clinical trial pre-specified a primary outcome measure that utilized a single efficacy variable, a composite outcome measure, or multiple outcome measures. In studies that utilized a single efficacy variable, only three activity domains were included: vitreous haze (eight of 18 clinical trials using an activity domain as a primary outcome measure); macular oedema (8 clinical trials); and treatment requirement (2 clinical trials). In clinical trials with either a composite primary efficacy outcome measure or multiple primary efficacy outcome measures, six additional activity domains were utilized: anterior chamber (AC) cells; AC flare; vitreous humour cells; vitreous “snow-balls”; chorioretinal inflammatory lesions; and retinovascular inflammation. In nine clinical trials, the outcome measure was listed as “activity”, but without specification of the domain(s) measured to define the measure. Vitreous haze was again the leading activity domain utilized for clinical trials with a composite primary outcome measure (30 of 32 clinical trials using an activity domain; *p* = 0.0002 vs. clinical trials with single study variables as outcome measures; Fisher exact test), but its use was significantly less common than in clinical trials using multiple primary outcome measures (2 of 20 clinical trials using an activity domain; *p* = 0.0265 vs. clinical trials with single study variables as outcome measures; Fisher exact test). Use of macular oedema was less common as an activity domain in clinical trials with a composite outcome measure (four of 32 clinical trials using an activity domain; *p* = 0.017 clinical trials with single study variables as outcome measures; Fisher exact test), but was used in a similar proportion of clinical trials with multiple primary outcome measures (seven of 20 clinical trials with an activity domain; no significant difference vs. clinical trials with single study variables as outcome measures). Visual acuity was the domain used to measure the dimension of visual function performance in all cases (Fig. 2).

The quality of pre-specified outcome measures was considered to be poor in 12 (12 %) 0f 104 clinical trials; for example, some used imprecise terms, such as “inflammation grade,” without specifying the variable to be measured.

## Discussion

There has been a lack of consensus with regard to the outcome measures that should be collected in clinical trials of efficacy for intermediate, posterior, and panuveitis. In this systematic review, we analysed the outcome measures used in all uveitis clinical trials included in all ICMJE-approved clinical trial registries (from the repository inception through to 01 October 2013). Considerable heterogeneity was noted, with at least 14 different domains being used as primary outcome measures. Additionally, although pre-specified primary outcome measures are required by these registries, the outcome measures were poorly defined in a substantial number of clinical trials, such that they provided inadequate information for reproducibility. Furthermore, we noted that 23 (22 %) of 104 clinical trials had registered multiple primary outcome measures, which is not recommended; if fact, the CONSORT statement on reporting of clinical trials specifically advises against doing so [18].

Before considering the challenging issue of outcome measure heterogeneity further, it is worth noting the specific outcome measures selected for the clinical trials that have been registered. For the purposes of this systematic review, we classified outcome measures into distinct dimensions: disease activity (e.g. vitreous haze score); disease-associated tissue damage or complications (e.g. cataract); visual function performance (e.g. high-contrast distance visual acuity); and patient-reported visual function (e.g. NEI-VFQ-25). Of the included studies that addressed efficacy, 74 % included one or more variables related to disease activity as primary outcome measures; 52 % included visual acuity as a primary outcome measure and 4 % included one or more variables of disease-associated tissue damage or complications as primary outcome measures. No studies included a measure of patient reported visual function as a primary outcome measure.

It may be argued that these dimensions reflect a disease pathway viewed from opposite ends by the clinician (who makes treatment decisions based primarily on disease activity) and the patient (whose primary concern is the impact of the disease on function and quality of life). All four dimensions are inter-related, but the relationship between them is complex. For example, increasing central macular thickness (CMT) due to macular edema is associated with worsening visual acuity, and worsening visual acuity is associated with worsening patient-reported visual function, but the relationship is not necessarily direct, and the correlation between them is not perfect [19–22].

The most common measures of disease activity used as primary outcome measures were vitreous haze and macular oedema. It is interesting to contrast these domains. Vitreous haze, as assessed using the NEI vitreous haze score, is a subjective measure, with a substantial interobserver variability (agreement, *k* = 0.53 for exact grade; *k* = 0.75 for within 1 grade), and as discussed earlier, is additionally limited by the narrow range seen in association with most forms of uveitis (scores of 0, 0.5+, 1+; less commonly 2+) [14, 15]. In contrast, macular oedema, as assessed by optical coherence tomography can provide objective measures of high reproducibility (e.g. automated measurement of CMT) which are highly sensitive to detecting change [22, 23]. It is important to recognize that these domains are measuring different aspects of the disease, and that many patients with vitreous inflammatory reactions will not have macular oedema and vice versa. The impact of OCT in the measurement of macular oedema does, however, highlight the value that objective quantification by imaging modalities (including, but not limited to OCT) might in the future bring to the assessment of vitreous inflammatory reactions, chorioretinitis, retinal vasculitis, and other manifestations of disease activity in intermediate, posterior, and panuveitis [8, 24].

It is noteworthy that all clinical trials that included measures of visual function performance, used high-contrast distance visual acuity. Although distance visual acuity is a standard assessment in almost all ophthalmic studies, it is increasingly recognized to be an imperfect indicator of day-to-day visual function [8]. Other components of visual function that might be considered include contrast sensitivity, reading acuity, reading speed, visual field sensitivity, and central retinal sensitivity [21, 25, 26]. The utility of determining these variables to assess the impact of uveitis on quality of life is not yet established, but their inclusion as secondary outcome measures in therapeutic clinical trials may provide valuable information in this regard.

The impact of altered visual function on quality of life may be objectified through patient reported outcome measures (PROM), such as the NEI-VFQ25 [27, 28]. The NEI-VFQ25 has been validated for patients with cataract, age-related macular degeneration, diabetic eye disease and glaucoma [28], but its validation among those with uveitis has been more limited [29]. Although the preferred PROM for most clinical trials related to uveitis has been the NEI VFQ-25, it is likely that not all of its questions are equally relevant to this population [29–31]. The HURON study reported that, although results for all questions differed significantly from the normal-vision population [32], only near-vision, distance vision, peripheral vision and social functioning questions showed significant change with treatment [33]. It is of interest that we found no uveitis clinical trials that included patient-reported variables as a primary outcome measures, even when multiple or composite outcome measures were used. It is important that those involved in the design of uveitis clinical trials recognize the value of such outcome measures in providing the patient perspective and capturing a more holistic response to a given treatment (and its side-effects) than is provided by more familiar outcome measures such as visual acuity. Although not the primary focus of this systematic review, we did note that patient-reported outcome measures (such as the NEI-VFQ25) are being used with increasing frequency as secondary outcome measures. The use of these secondary outcome measures is likely to provide additional, valuable information that will help inform patients, clinicians, and other stake-holders, as to the broader benefit of a therapeutic agent under consideration.

The field of uveitis is not alone in facing the problem of heterogeneous outcome measures in drug development. In a survey of 2000 trials dealing with schizophrenia, Thornley and Adams found that 640 different instruments had been used, of which 369 had been used only once [34]. In choosing outcome measures, CONSORT strongly encourages the use of “previously developed and validated scales or consensus guidelines … both to enhance quality of measurement and to assist in comparison with similar studies” [18]. Doing so matters because heterogeneity of outcome renders comparison of clinical trials and meta-analyses difficult or even impossible. In rare diseases, where the number of trials will always be more limited, it is even more important that there is consensus regarding the selection of outcome measures so that such evidence can be gathered to inform patients, clinicians, regulators and healthcare funders.

While our systematic review does not attempt to provide the solution to outcome heterogeneity in the study of uveitis, it does provide an estimate of the scale of the problem and provides data to inform this important debate. The variation in outcome measures chosen by the investigators of these 104 clinical trials is, in itself, an indicator that there is likely to be no easy answer to the problem. Approaches to finding a solution may need to face the “lumping vs. splitting” dichotomy among uveitis specialists. For example, Behcet disease, pars planitis syndrome, birdshot chorioretinopathy, and Vogt-Koyanagi-Harada disease are distinct forms of uveitis, with unique signs of inflammation, yet all have, in the past, ended-up in common clinical trials that use the same outcome measures. The risk is that one may fail to detect therapeutic benefit due to the high level of “noise” introduced by the amalgamation of too-wide a range of clinical entities for two reasons. First, this grouping is based on a taxonomy which reflects anatomy rather than aetiology, and so it cannot be assumed that a particular therapy will be equally efficacious across all uveitis syndromes within the same group. Second even if a drug were to be effective across multiple syndromes (due to overlapping pathogenetic pathways), there may be no single outcome measure that can adequately detect a positive response in all these different syndromes, each of which has a unique phenotype. The option of syndrome-specific clinical trials has not been possible, despite making “biological sense”, because of logistic challenges, particularly around recruitment. There is also a pragmatic issue with disease-specific clinical trials: the narrower in scope the population within a clinical trial, the narrower any subsequent regulatory approval will be.

Others have tackled the issue of heterogeneous outcome measures in clinical trials by establishing “core outcome sets” (COS). This approach provides a standardized set of outcome measures that are reported in all clinical trials of a condition under consideration, while still allowing the investigator discretion to choose his or her own primary or secondary outcome measures [35]. The use of COS may enhance evidence synthesis by reducing heterogeneity (shared outcome measures), reducing outcome-reporting bias (as the whole COS is reported) and improving the statistical power of any meta-analysis (more studies can be included). COS development is supported by a number of initiatives, such as COMET (Core Outcome Measures in Effectiveness Trials) and has been endorsed by the Cochrane Library, the GRADE (Grading of Recommendations Assessment, Development and Evaluation) working group, and the WHO [35, 36].

Another strategy relevant to this debate is the use of a composite outcome measure. While it may be argued that such outcome measures provide a more “holistic” assessment of a patient’s state (they frequently include visual acuity), it is likely that the frequent use of composite measures in the clinical trials that we reviewed (35 of 94 clinical trials with an efficacy measure) is driven by the lack of a single outcome measure suitable for all patients. This is supported by the observation that of these 94 clinical trials, 75 included a broad range of disorders (intermediate, posterior, and panuveitis), with only 18 being limited to a single disorder (and one study including two disorders). It should be noted that the design, use, and interpretation of composite endpoints is a challenging area, and has led to the FDA to put strict guidance in place as to their usage [37].

It is also important to consider how study variables are measured [35]. Although it was not the primary focus of this review, we noted considerable heterogeneity with regard to how a number of domains were measured. For example, visual acuity varied between clinical trials with regard to (1) measurement instrument (i.e. Snellen or the ETDRS chart); and (2) quantification (e.g. “improvement in 2 or more lines of Snellen visual acuity” or “improvement in LogMAR” by a specified number of letters). Similarly, the use of the NEI vitreous haze score varied with regard to quantification (number of steps required to be significant), and in some trials an additional scoring point (1.5+) was added.

We chose to limit our review to clinical studies in ICMJE-approved registries, for a number of reasons. It identifies all studies in which the investigators have pre-specified primary outcome measures and trial design; it reduces publication bias; and it provides a more current perspective on trial design than provided by published articles, most of which do not describe the full protocol, as it existed prior to commencement of the clinical trial.

We conducted the analysis on an ‘intention-to-trial’ basis; all registered clinical trials were included, regardless of whether or not they were later withdrawn, failed to recruit participants, or completed recruitment, but were never published. We felt that doing so was important, as there is substantial publication bias around clinical trials that fail to demonstrate a desired therapeutic benefit. This approach also ensured that we identified what the trialists perceived to be the most appropriate primary outcome measures at the time of trial design, rather than at the time of publication, thereby avoiding the publication bias that may have arisen from investigators selecting those outcome measures that provided a significant result instead of those that were pre-specified.

We recognise that our systematic review omitted a number of older studies of uveitis that predated the ICMJE requirements for registration. In its September 2004 editorial, the ICMJE announced that it would not consider a trial for publication unless the clinical trial had been included in an approved registry. Registration had to be undertaken prospectively (i.e. prior to patient enrolment) for any clinical trial starting enrolment after July 1, 2005. For clinical trials that began enrolment prior to that date, registration had to occur by September 13, 2005. Our review should therefore have a complete record of relevant clinical trials from the past 8 years, and due to retrospective registration in 2005, a number of additional clinical trials extending back as far as 2001.

In summary our systematic review formally surveys the heterogeneity present around outcome measures in recent and current clinical trials related to intermediate, posterior, and panuveitis. It does not address the issue of outcome measures for anterior uveitis, an important form of disease, but one that has not been the subject of many therapeutic clinical trials to date. We have reported that current clinical trial designs in uveitis prioritize clinician-observed measures of disease activity and objective measurements of visual function, and that patient-reported outcome measures did not feature as primary outcome measures in any registered clinical trial to date. We argue that the challenging issue of outcome measure selection for clinical trials of efficacy related to uveitis needs to be addressed, and that the uveitis community needs to work towards a new consensus regarding an approach to the use of outcome measures in therapeutic clinical trials involving patients with uveitis.
